# Neurodevelopment at Two Years in Preterm Infants: Corrected Versus Chronological Age

**DOI:** 10.3390/children13020219

**Published:** 2026-02-04

**Authors:** Barbara Caravale, Valentina Focaroli, Elvira Caramuscio, Cristina Zitarelli, Francesco Pisani, Corinna Gasparini, Paola Ottaviano, Antonella Castronovo, Marzia Paoletti, Daniela Regoli, Lucia Dito, Gianluca Terrin, Rosa Ferri

**Affiliations:** 1 Department of Developmental and Social Psychology, Sapienza University of Rome, 00185 Rome, Italy; cristina.zitarelli@uniroma1.it; 2 Department of Economic, Psychological, Communication, Educational and Motor Sciences, Università degli Studi Niccolò Cusano, 00166 Rome, Italy; valentina.focaroli@unicusano.it; 3 Department of Dynamic and Clinical Psychology and Health Studies, Sapienza University of Rome, 00185 Rome, Italycori.gaspar@gmail.com (C.G.); rosa.ferri@uniroma1.it (R.F.); 4 Department of Human Neuroscience, Azienda Ospedaliero-Universitaria Policlinico Umberto I, Sapienza University of Rome, 00185 Rome, Italy; francesco.pisani@uniroma1.it (F.P.); paola.ottaviano@uniroma1.it (P.O.);; 5 Department of Neonatology and NICU, S. Eugenio Hospital, 00144 Rome, Italy; 6 Department of Maternal and Child Sciences and Urology, Azienda Ospedaliero-Universitaria Policlinico Umberto I, Sapienza University of Rome, 00161 Rome, Italygianluca.terrin@uniroma1.it (G.T.)

**Keywords:** preterm infants, neurodevelopment, age correction, Bayley-III scales

## Abstract

**Highlights:**

**What are the main findings?**

- At 2 years, corrected age reveals selective developmental vulnerabilities, whereas chronological age indicates a generalised delay.
- Greater prematurity is associated with poorer developmental performance.

**What are the implications of the main findings?**

- Corrected age should be used at least until 24 months in the developmental assessment of pre-term children.
- Age correction should be tailored according to gestational age and developmental domain to im-prove clinical accuracy.

**Abstract:**

Background: Preterm birth is a significant risk factor for neurodevelopmental delays, but the appropriate use and timing of age correction for developmental assessment remain debated. Objective: This study investigated psychomotor development in preterm children at two years of age, with the aim of clarifying whether age correction remains necessary at this stage, particularly across different gestational age groups. Methods: A total of 161 preterm infants were assessed at a mean chronological age of 25.4 months (mean corrected age: 23.3 months) and compared with two control groups of typically developing children matched for gender and either corrected age (Control–Corr, *N* = 88) or chronological age (Control–Chron, *N* = 87). The preterm group was further stratified by gestational age: extremely preterm (<28 weeks), very preterm (28–31 weeks), and moderate-to-late preterm (32–36 weeks). Cognitive, Language (Receptive, Expressive), and Motor (fine, gross) scales of Bayley-III were analysed using *t*-tests and MANOVAs. Results: Using corrected age, preterm children showed a selective profile, with deficits in Receptive Language, borderline mean score in Gross Motor, and preserved performance in Cognitive, Expressive Communication, and Fine Motor. When compared with controls of the same age, significant differences emerged in the Cognitive, Language, and Gross Motor, but not Fine Motor, domains. In contrast, scoring by chronological age produced a generalised delay, with preterm children performing significantly worse than chronological-age controls across all domains. Subgroup analyses further showed that extremely preterm children already displayed marked Language vulnerabilities at corrected age, which became more severe with chronological scoring and extended to other domains. Very preterm children also fell into the deficit range in Cognitive, Language, and Gross Motor scales/subscales when chronological age was applied, whereas moderate-to-late preterm children performed comparatively better. Conclusions: Developmental assessment using corrected age remains essential at least until 24 months, especially for extremely and very preterm children, to avoid substantial overestimation of developmental difficulties. Chronological scoring, while helpful to highlight persistent vulnerabilities, may inflate delay classification if used too early. Tailoring correction strategies by gestational age and developmental domain could provide a more accurate and clinically meaningful representation of preterm children’s developmental trajectories.

## 1. Introduction

Preterm birth, defined as delivery before 37 completed weeks of gestation, accounts for approximately 10% of live births worldwide, representing one of the leading causes of neonatal morbidity and mortality [1]. Although advances in perinatal and neonatal care have significantly improved survival rates, children born preterm, especially those with lower gestational age and birth weight, remain at elevated risk for a wide range of neurodevelopmental impairments and neurological disorders [2,3,4]. These include language disorders, developmental coordination disorder, intellectual disability, attention-deficit/hyperactivity disorder, autism spectrum disorder, and later learning disabilities. Notably, these conditions may follow different developmental trajectories, with early vulnerabilities often emerging in Cognitive, Language, and Motor domains during the first years of life. While some impairments can be detected within early childhood, others may only become evident later, during school age, adolescence, or even adulthood [5].

Given the increased vulnerability associated with preterm birth, numerous national and international guidelines recommend early neurodevelopmental assessment to support the timely identification of delays and facilitate access to appropriate interventions [6,7,8,9]. Within this framework, two years of age represents a key developmental phase in early childhood, during which Cognitive, Language, and Motor abilities show increasing organisation and stability. At this stage, early neurodevelopmental vulnerabilities become more clearly identifiable, and developmental assessments increasingly inform clinical decision-making, follow-up strategies, and early intervention planning.

The Bayley Scales of Infant and Toddler Development, Third Edition (Bayley-III) [10], is one of the most widely used instruments worldwide for the early assessment of Cognitive, Language, and Motor development, and several studies support its predictive validity for later outcomes, particularly in Cognitive and Language domains [11,12,13].

The use of the Bayley-III is also well established in the Italian clinical setting, where it has been adopted in follow-up programmes for preterm infants and recognised as a reference tool for identifying early developmental vulnerabilities and planning targeted interventions [14,15,16].

Developmental assessments are conventionally standardised based on a child’s chronological age. For infants born preterm, however, corrected age, calculated by subtracting the number of weeks of prematurity from chronological age, is commonly applied to provide a more accurate comparison with term-born peers. In clinical practice, correction is essential during the first year of life and generally applies up to 24 months. This recommendation is supported by both the American Academy of Pediatrics and the Bayley-III manual [10,17].

The earlier literature (1980s–1990s) consistently showed that corrected age better reflected developmental outcomes than chronological age during infancy. Palisano (1986) [18] demonstrated significant differences in Motor scores between corrected and chronological age up to 18 months, while other longitudinal studies reported that discrepancies could persist into the preschool years [19,20]. These findings established corrected age as the more accurate reference point in the early assessment of preterm infants.

The more recent literature supports a flexible, individualised approach to age correction. Some authors advocate extending correction beyond 24 months, particularly for extremely preterm children or those with persistent developmental difficulties. For example, Aylward (2020) [21] noted that failing to correct assessments of Cognitive, Language, and Motor skills up to 3 years of age places preterm infants at a clear disadvantage, while D’Agostino (2010) [22] suggested that in the most immature infants, correction may need to be prolonged even further. In addition, recent findings indicate that age correction can still influence developmental assessment at school age, especially in higher-order domains such as executive functions [23], further underscoring the uncertainty surrounding the optimal duration of its use.

Other authors have instead proposed a domain-specific approach. For instance, Morsan et al. (2018) [24] examined developmental patterns at 12 months of age. They found that corrected age provided a more accurate estimate of Cognitive performance, while chronological age better reflected Motor outcomes, and Language results remained inconclusive. Whether such domain-specific differences extend into later developmental stages remains unclear, as the study was limited to infancy.

These perspectives illustrate that there is no single, universally applicable strategy for age correction, and both approaches have important implications. While corrected age helps avoid overestimating developmental delay in early assessments, prolonged correction may risk masking actual difficulties, potentially delaying access to early intervention services [25]. Conversely, relying solely on chronological age can artificially lower test scores, increasing rates of developmental delay classification and causing unnecessary concern for families [26,27]. Overall, the literature shows a broad consensus that correction should be applied throughout the first year of life. Beyond this point, however, there is no general agreement on the optimal use of age correction during the second year of life. This uncertainty has important implications for both clinical practice and the interpretation of neurodevelopmental outcomes at two years of age.

Despite these ongoing debates, few studies have directly compared preterm children’s developmental profiles using corrected and chronological age with those of typically developing peers across multiple domains using the Bayley-III [24]. The present study aims to address this gap by systematically evaluating the developmental outcomes of a large cohort of preterm infants at approximately 24 months of age. Their scores were compared with those of two control groups of typically developing children matched for gender and either corrected age or chronological age. Additionally, the study explores whether age correction appears more appropriate for certain gestational age groups than others at 24 months.

## 2. Methods

### 2.1. Participants

Participants were recruited between 2018 and 2024 through the clinical neurodevelopmental follow-up programme for preterm infants at the Policlinico Umberto I Hospital (Rome, Italy). Inclusion criteria were birth before 37 completed weeks of gestation, discharge from the Neonatal Intensive Care Unit of Policlinico Umberto I Hospital, and enrolment in the routine neurodevelopmental follow-up programme with assessment at approximately 24 months of age. Exclusion criteria were major congenital malformations, genetic syndromes, severe sensory impairments (e.g., blindness or deafness), and severe neuromotor impairment preventing standardised assessment.

#### 2.1.1. Preterm Group

This study included 161 preterm infants (PRE group; 84 females, 77 males). At the time of assessment, the mean chronological age was 25.43 ± 1.17 months, and the mean corrected age was 23.26 ± 1.22 months.

All children were assessed using the Italian version of Bayley-III [28], and two sets of developmental scores were obtained. The first set, referred to as PRE–Corr, was based on the children’s corrected age, with raw scores converted using the normative data corresponding to a mean corrected age of approximately 23 months. The second set, referred to as PRE–Chron, was based on the children’s chronological age, using normative data appropriate for a mean chronological age of approximately 25 months.

To further explore developmental outcomes related to prematurity severity, the PRE group was stratified according to gestational age at birth, following the World Health Organization classification [29]: extremely preterm (<28 weeks), very preterm (28–31 weeks), and moderate-to-late preterm (32–36 weeks and 6 days).

#### 2.1.2. Control Groups

The PRE group was compared to two control groups (Control–Corr and Control–Chron) of typically developing children drawn from the Italian standardisation sample of the Bayley-III. These control groups were matched to the preterm group in a 2:1 ratio based on age and gender. The Control–Corr group (*N* = 88) was matched to the PRE–Corr group using corrected age (M = 23.39, SD = 1.42; t(247) = −0.778, *p* = 0.431; Cohen’s d = −0.104), while the Control–Chron group (*N* = 87) was matched to the PRE–Chron group using chronological age (M = 25.31, SD = 1.30; t(246) = 0.730, *p* = 0.466; Cohen’s d = 0.097). Matching criteria prioritised age (corrected or chronological, depending on the comparison) and gender to ensure comparability across developmental domains.

### 2.2. Procedures

Preterm infants were enrolled through the clinical follow-up programme for preterm births at the Neonatal Intensive Care Unit (NICU) of Policlinico Umberto I Hospital, Rome, Italy. Psychomotor development was evaluated using the Italian adaptation of the Bayley-III [28], administered by trained professionals during routine neurological follow-up visits scheduled at six-month intervals throughout the first two years of life. All evaluations were conducted in a child-friendly setting to promote optimal engagement and performance.

The research protocol was approved by the Regional Ethics Committee of Lazio (Ref. number 7471, Prot. 0168/2024), and written informed consent was obtained from all parents or legal guardians prior to participation.

### 2.3. Measures

The Bayley-III assesses five developmental domains, of which only Cognitive, Language, and Motor were analysed in the present study. The Language domain comprises two subscales, Receptive Language (RL) and Expressive Language (EL), while the Motor domain includes Fine Motor (FM) and Gross Motor (GM) subscales. Each main domain yields a composite score (M = 100, SD = 15), and each subscale provides a scaled score (M = 10, SD = 3), enabling a detailed evaluation of specific developmental areas. In line with common clinical conventions, scaled scores between 7 and 8 were considered indicative of borderline performance, while scores below 7 were classified as deficit-level.

### 2.4. Data Analysis

Statistical comparisons were planned a priori and were hypothesis-driven. Effect sizes were reported alongside *p*-values to support interpretation of the results. Group differences on the Bayley-III composite and scaled scores were analysed using independent-samples *t*-tests. Comparisons were conducted between preterm children and the two control groups based on corrected age (PRE–Corr vs. Control–Corr) and chronological age (PRE–Chron vs. Control–Chron).

Effect sizes were calculated using Cohen’s d and interpreted according to Hyde’s [30] guidelines: small (0.11–0.35), moderate (0.36–0.65), large (0.66–1.00), and very large (>1.00).

A one-way MANOVA with planned simple contrasts was conducted comparing the E-PRE, V-PRE, and ML-PRE subgroups to examine developmental outcomes by gestational age. Both corrected and uncorrected scores were analysed. Partial eta squared (η^2^) was used to report effect sizes, classified as small (η^2^ = 0.01), medium (η^2^ = 0.06), or large (η^2^ = 0.14).

One-way ANOVAs with planned simple contrasts were also conducted to compare extremely preterm (E-PRE), very preterm (V-PRE), and moderate-to-late preterm (ML-PRE) subgroups considering neonatal characteristics like birth weight (g), 5′ Apgar score and length of NICU stay (days).

## 3. Results

Descriptive characteristics of the study groups are presented in Table 1, including sample size, gender distribution, and ages (M ± SD) for the preterm and control groups, with the preterm group further subdivided by gestational age. Table 2 reports neonatal variables for the preterm group, including birth weight, 5-min Apgar score, and length of hospital stay. As expected, the three gestational age subgroups differed on these variables, with the extremely preterm group showing lower birth weight, lower Apgar scores, and longer NICU stays.

In the PRE–Corr group, mean scores indicated deficit-level performance in Receptive Communication (mean value below seven) and vulnerability in Cognitive, Expressive Communication, and Gross Motor skills, with mean values close to eight, while Fine Motor performance was within the expected range (Table 3).

Comparisons with the Control–Corr group, matched for corrected age at approximately 23 months, revealed significantly lower scores in the PRE–Corr group across Cognitive, Receptive Communication, Expressive Communication, and Gross Motor domains. Fine Motor skills did not differ between groups. Overall, these findings indicate that, relative to children matched for corrected age (mean 23 months), the PRE–Corr group showed reduced performance in Cognitive, Language, and Gross Motor domains, whereas Fine Motor abilities were comparable.

In the PRE–Chron group, mean scores indicated deficit-level performance in Cognitive, Receptive Communication, and Gross Motor (all means <7), and borderline performance in Expressive Communication and Fine Motor (both just above 7) (Table 4). Comparisons with the Control–Chron group, matched for chronological age at approximately 25 months, revealed significantly lower scores in the PRE–Chron group across all Bayley-III subscales, including Cognitive, both Receptive and Expressive Communication, and both Fine and Gross Motor domains. Overall, these findings show that, relative to children matched for chronological age of 25 months, the PRE–Chron group performed worse across all developmental domains assessed by the Bayley-III.

**Figure 1:**
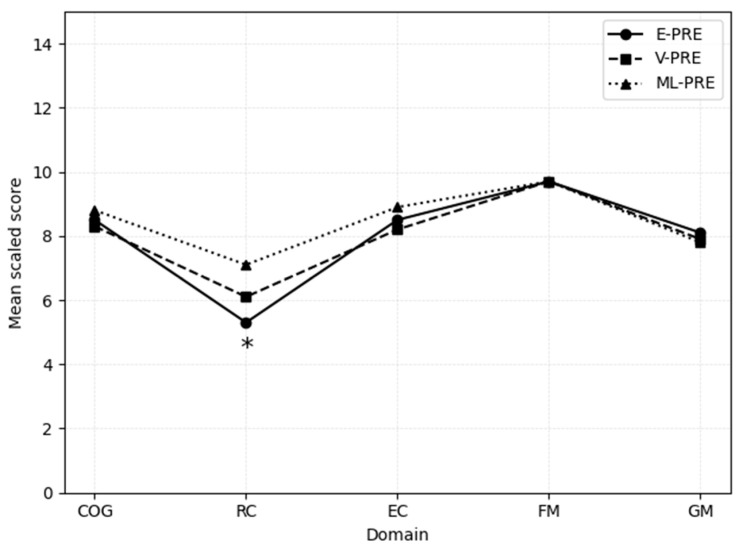
Developmental performance profiles (Bayley-III scales) of the E-PRE, V-PRE, and ML-PRE subgroups based on corrected age scores (PRE–Corr group). Mean scaled scores are shown for Cognitive (COG), Receptive Communication (RC), Expressive Communication (EC), Fine Motor (FM), and Gross Motor (GM) scaled scores. Note: The E-PRE group obtained a significantly lower mean score than the ML-PRE group in the Receptive Communication subscale (*p* = 0.025). No significant differences were found among the Cognitive, Expressive Communication, Fine Motor, or Gross Motor scale/subscales subgroups. * indicates a statistically significant difference in scores (*p* < 0.05).

A further analysis compared the three preterm subgroups, E-PRE, V-PRE, and ML-PRE, using corrected age scores. As shown in Figure 1, the only significant difference emerged in the Receptive Communication subscale: the E-PRE group obtained a markedly lower mean score (M = 5.4, SD = 3.2) compared to the ML-PRE group (M = 7.1, SD = 2.9; *p* = 0.025). Conversely, no significant differences were found among the subgroups in Cognitive (E-PRE: M = 8.4; V-PRE: M = 8.2; ML-PRE: M = 8.8), Expressive Communication (8.5, 8.2, 8.9), Fine Motor (9.6, 9.7, 9.6), and Gross Motor (8.0, 7.8, 7.7) performance.

**Figure 2:**
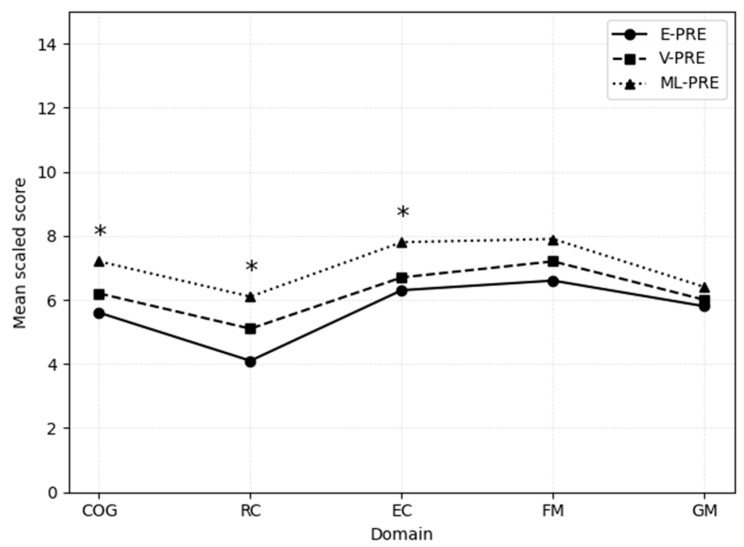
Developmental performance profiles of the E-PRE, V-PRE, and L-PRE subgroups (uncorrected age scores). Note: The ML-PRE group obtained significantly higher scores than both the E-PRE and V-PRE groups on the Cognitive scale (*p* = 0.008) and on Receptive Communication (*p* < 0.001) and Expressive Communication (*p* = 0.013). On the Fine Motor scale, the ML-PRE group scored higher than the E-PRE group, but this difference did not reach significance (*p* = 0.077). No significant differences emerged among the three subgroups in the Gross Motor scale. * indicates a statistically significant difference in scores (*p* < 0.05).

Finally, the three PRE subgroups were compared using chronological age scores. As shown in Figure 2, the ML-PRE group obtained significantly higher scores than both the E-PRE (5.6 ± 2.84) and V-PRE (6.3 ± 2.49) groups on the Cognitive scale (ML-PRE: 7.3 ± 2.60; *p* = 0.008) and on Receptive Communication (E-PRE: 4.1 ± 2.35; V-PRE: 5.2 ± 2.53; ML-PRE: 6.2 ± 2.49; *p* < 0.001) and Expressive Communication (E-PRE: 6.3 ± 2.89; V-PRE: 6.7 ± 2.31; ML-PRE: 7.8 ± 2.59; *p* = 0.013). On the Fine Motor scale, the ML-PRE group (7.9 ± 2.61) scored higher than the E-PRE group (6.6 ± 3.17), but this difference did not reach significance (*p* = 0.077). No significant differences emerged among the three subgroups in the Gross Motor scale (E-PRE: 5.8 ± 1.68; V-PRE: 6.0 ± 1.87; ML-PRE: 6.4 ± 1.93).

Overall, developmental test results do not constitute formal diagnoses but provide a standardised characterisation of neurodevelopment and support the clinical interpretation of developmental functioning.

## 4. Discussion

The present study analysed developmental skills of preterm infants at 2 years of age, considering two control groups of typically developing peers: one matching for corrected age and the other for chronological age. Indeed, the main aim was to provide empirical evidence to the ongoing debate on whether and how long age correction should be applied in developmental assessment. Accounting for prematurity remains a critical methodological issue in developmental research, as preterm infants often exhibit early delays in Cognitive, Language, and Motor domains [31,32]. This approach recognises that central nervous system maturation follows a biologically determined course aligned with gestational age, approximating the developmental timeline that would have occurred if the infant had been born at term.

Before discussing the specific developmental patterns observed, it is important to emphasise that the profiles identified in the present study do not represent formal diagnoses of neurodevelopmental disorders. Rather, they reflect early patterns of Cognitive, Language, and Motor functioning that are clinically meaningful and eventually associated with an increased risk of later neurodevelopmental disorders.

In our group of preterm children, mean scores based on corrected age revealed a deficit specifically in Receptive Communication. In contrast, Cognitive, Expressive Communication, and Gross Motor scores fell within the borderline range, and Fine Motor abilities were within the expected limits. This profile suggests that developmental weaknesses appear selective rather than generalised when corrected age is around 23 months. The comparison with the control group of children of approximately 23 months (Control–Corr group) was consistent with this pattern. Significant differences emerged in Receptive and Expressive Communication and Gross Motor domains, while Cognitive and Fine Motor performance did not differ. Thus, corrected age appears to mitigate developmental gaps and reduce the risk of overestimating general delay, which aligns with prior studies [33]. Importantly, our finding that Language is a weak domain aligns with previous evidence showing that language difficulties are particularly prevalent in preterm children across infancy [34] and the second year of life [35,36]. In addition, our results highlight Motor development, particularly Gross Motor skills, as another area of vulnerability, according to the literature indicating that motor difficulties often persist beyond the early years [37,38]. Together, these findings suggest that from the second year of life, preterm children do not show a global developmental delay but rather selective fragilities in specific domains, with such difficulties often persisting into the preschool years [20,39]. Extending this perspective, evidence from older cohorts indicates that the issue of age correction may remain relevant even at school age: Wehrle et al. [23], for instance, showed that in very preterm children at school age, differences in executive functions compared to full-term peers were significantly reduced when corrected age was considered, suggesting that, at least for higher-order neurocognitive domains, the benefits of age correction may extend well beyond early childhood.

However, the profiles identified in this study reflect a cross-sectional snapshot of development at approximately 24 months of age. While selective weaknesses are evident at this stage, the present data do not allow conclusions about their evolution in later childhood, which will require longitudinal follow-up.

By contrast, when chronological age was used to score the Bayley-III (around 25 months), mean scores in the preterm group dropped below seven in multiple domains, including Cognitive, Receptive Communication, and Gross Motor, with additional vulnerability in Expressive Communication and Fine Motor. This pattern indicates a more generalised developmental lag across domains. The comparison with the Control–Chron group reinforced this interpretation, showing significant differences across all Bayley-III subscales. These findings highlight how chronological age heightens developmental discrepancies and raises the classification of delays [26,27].

The analysis of gestational age subgroups offered further insight. When corrected age was used (around 23 months of age), extremely preterm children showed markedly low mean scores in Receptive Communication. In contrast, the very preterm and moderate-to-late preterm groups performed closer to the expected range. On the other hand, when chronological age norms were applied (around 25 months of age), deficits became more widespread: extremely and very preterm children scored in the clinical range across multiple domains, with dramatic values in Receptive Communication among the extremely preterm subgroups. Regarding between-group comparisons, at corrected age, only one significant difference was observed, with extremely preterm children performing worse than moderate-to-late preterm children in Receptive Communication. However, when chronological age was applied, the differences became more pronounced: the moderate-to-late preterm group outperformed extremely and very preterm children across Cognitive and Language domains and performed better than the extremely preterm group in Fine Motor skills.

Taken together, these results indicate that extremely preterm children already show specific vulnerabilities at 23 months corrected age, particularly in Receptive Communication, while very preterm and moderate-to-late preterm children perform closer to the expected range. However, when chronological age norms are applied, differences between subgroups become more evident: extremely and very preterm children show lower performance than moderate-to-late preterm peers across Cognitive and Language subscales, with additional weaknesses in Fine Motor skills for the extremely preterm group.

Comparisons among gestational age subgroups confirmed that Language was the most fragile domain, and that under chronological scoring, developmental gaps extended across multiple domains.

For these reasons, age correction should be maintained at least until 24 months of corrected age, particularly in extremely and very preterm children, to accurately characterise the child’s developmental profile and allow strengths and vulnerabilities to emerge. At this stage, direct comparison with chronological-age peers may accentuate apparent delays, contribute to a flattening of developmental scores, and increase the risk of over-classification of generalised developmental delay rather than reflecting the child’s underlying developmental organisation. For moderate-to-late preterm children, correction may be less critical, but an individualised approach is advisable to prevent excessive diagnoses of developmental disorders.

These findings also underscore the importance of gestational age as a predictor of outcome, in line with studies documenting persistent difficulties in extremely preterm children. Indeed, evidence from both classic and more recent studies shows that extremely preterm infants score lower than their less immature peers from the first years of life onwards [20,39,40,41], reinforcing the view that the degree of prematurity is a key determinant of early developmental trajectories, with the most immature infants showing the broadest and most enduring challenges.

In this context, it should also be noted that the present sample reflects a real-world neurodevelopmental follow-up population, including preterm infants with neonatal complications who were clinically stable at assessment. This is important when interpreting developmental variability, particularly in extremely and very preterm groups.

Our study also invites reflection on the potential usefulness of chronological age scoring. While it tends to accentuate developmental gaps, this perspective can be clinically informative, as it reflects children’s everyday life expectations. Corrected age provides a fairer estimate of developmental potential, whereas chronological age offers insight into children’s concrete challenges in family routines and educational contexts. This perspective can also guide rehabilitation, helping professionals to define therapeutic objectives that are realistic for the child’s age and daily environment, while still aligned with their developmental potential.

Several limitations of this study should be acknowledged. First, the clinical group and the two control groups, drawn from the Bayley-III normative sample, were matched only by age and gender. This limited control over background variables such as socioeconomic status, parental education, and regional variation may have impacted the developmental outcomes observed. Second, neonatal variables such as birth weight, Apgar score, and length of NICU stay were reported descriptively but were not included as covariates in the developmental analyses. As the primary aim of the study was to compare age-based scoring approaches rather than to estimate the independent contribution of neonatal risk factors, covariate-adjusted models (e.g., ANCOVA or regression) were not applied. This methodological choice limits causal inference and should be considered when interpreting the findings. Third, developmental outcomes were evaluated only at about 24 months of age, and it remains uncertain whether the observed patterns extend into later childhood. Moreover, the cross-sectional design prevents conclusions about developmental trajectories over time. Finally, the distribution of participants across gestational age subgroups was uneven, with smaller numbers in the extremely preterm category, which may have decreased the statistical power to detect subgroup differences.

Despite these limitations, our findings indicate that the choice between corrected and chronological age remains critical at two years of age. Corrected age offers a fairer estimate of developmental potential, highlighting selective weaknesses mainly in Language and Motor skills, whereas chronological age portrays a more global delay. For extremely and very preterm infants, maintaining correction until at least 24 months appears essential, whereas for moderate-to-late preterm children, a more flexible, individualised approach may be sufficient. Future longitudinal studies following children into school age are needed to determine whether the advantages of age correction last as neurocognitive demands increase over time and to inform best practices in assessment and intervention.

Declaration of generative AI and AI-assisted technologies in the writing process. During the writing process of this work, the authors used AI-assisted technology (GPT-5) to improve the readability and language of the manuscript. After using this tool, the authors carefully reviewed and edited the content as needed. The authors take full responsibility for the content of the published article.

## Figures and Tables

**Table 1:** Sample characteristics: size (*N*), gender distribution, and ages (mean ± SD) for clinical and control groups.

	Total *N*	Females *N*	Males *N*	Chronological Age (Months) M (SD)	Corrected Age (Months) M (SD)
Preterm group (PRE)	161	84	77	25.43 (1.17)	23.26 (1.22)
E-PRE (<28 wks)	30	18	12	25.92 (1.24)	22.96 (1.30)
V-PRE (28–31 wks)	68	28	40	25.46 (1.16)	23.13 (1.27)
ML-PRE (32–36 wks)	63	33	30	25.16 (1.07)	23.53 (1.11)
Control groups Control–Corr	88	44	44	23.39 (1.42)	—
Control–Chron	87	42	45	25.31 (1.30)	—

Note: E-PRE, V-PRE, and ML-PRE are subgroups of the PRE group based on gestational age. Control–Corr = control group matched on corrected age. Control–Chron = control group matched on chronological age.

**Table 2:** Preterm-group neonatal characteristics.

	Total PRE Group (*N* = 161)	Extreme (E) (*N* = 30)	Very (V) (*N* = 68)	Moderate/Late (ML) (= 63)	*p*	η^2^ *	Planned Contrast
Birth weight (g) M ± SD [range]	1418.7 ± 394.2 [1350.9–1486.6]	1079.6 ± 202.4 [992.1–1167.1]	1369.29 ± 381.4 [1269.9–1468.7]	1633.1 ± 348 [1534.2–1732]	<0.001	0.250	[E] < [V] < [ML]
5′ Apgar score M ± SD [range]	6.8 ± 1.6 [7.5–8]	7.7 ± 1.4 [6.24–7.42]	7.7 ± 1.2 [7.3–8]	8.3 ± 1.4 [7.9–8.6]	<0.001	0.134	[E] < [V] < [ML]
Length of stay (days) M ± SD [range]	52 ± 27.1 [47.47–56.45]	76.2 ± 25 [66.65–85.69]	56 ± 24.3 [49.7–62.7]	35.6 ± 18.9 [30.4–40.5]	<0.001	0.324	[E] > [V] > [ML]

* η^2^ = 0.01 for small effect, 0.06 for medium effect, and 0.14 for large effect.

**Table 3:** Comparisons between PRE–Corr group (*N* = 161) and Control–Corr group (23 months) (*N* = 88) on Bayley-III subscales (corrected age scores).

Bayley-III Subscale	PRE–Corr (M ± SD)	Control–Corr (M ± SD)	Mean Diff.	t	df	*p*	Cohen’s d ^¥^	95% CI
COG	8.46 ± 2.95	9.53 ± 3.14	−1.07	−2.68	247	0.008	−0.36	[−0.62, −0.09]
RC	6.45 ± 2.99	9.89 ± 2.98	−3.44	−8.68	247	<0.001	−1.15	[−1.43, −0.87]
EC	8.53 ± 2.90	9.65 ± 2.97	−1.11	−2.87	247	0.004	−0.38	[−0.64, −0.12]
FM	9.61 ± 3.03	9.43 ± 3.30	+0.18	0.44	247	0.660	0.06	[−0.20, 0.32]
GM	7.81 ± 2.25	9.84 ± 3.16	−2.03	−5.35	137 #	<0.001	−0.78	[−1.05, −0.51]

Note: COG = Cognitive; RC = Receptive Communication; EC = Expressive Communication; FM = Fine Motor; GM = Gross Motor. **^¥^** Hyde’s guidelines [30]: small effect (0.11 < Choen’s d < 0.35); moderate effect (0.36 < Choen’s d < 0.65), large effect for (0.66 < Choen’s d < 1.00), or very large effect (d > 1.00). # Welch’s *t*-test.

**Table 4:** Comparisons between the PRE–Chron group (*N* = 161) and the Control–Chron group (25 months) (*N* = 87) on Bayley-III subscales (chronological age scores).

Bayley-III Subscales	PRE–Chron (M ± SD)	Control–Chron (M ± SD)	MeanDiff.	t	df	*p*	Cohen’s d ^¥^	95% CI
COG	6.57 (2.66)	10.08 (2.89)	−3.51	−9.59	246	<0.001	−1.28	[−1.56, −0.99]
RC	5.38 (2.58)	9.68 (2.65)	−4.30	−12.69	246	<0.001	−1.69	[−1.99, −1.38]
EC	7.05 (2.59)	9.74 (2.62)	−2.69	−7.76	246	<0.001	−1.03	[−1.31, −0.76]
FM	7.40 (2.71)	9.89 (2.97)	−2.49	−6.67	246	<0.001	−0.89	[−1.16, −0.61]
GM	6.12 (1.87)	9.69 (2.85)	−3.57	−10.53	126.94 #	<0.001	−1.58	[−1.87, −1.28]

Note: COG = Cognitive scale; RC = Receptive Communication scale; EC = Expressive Communication scale; FM = Fine-Motor scale; GM = Gross-Motor scale. **^¥^** Hyde’s guidelines [30]: small effect (0.11 < Choen’s d < 0.35); moderate effect (0.36 < Choen’s d < 0.65), large effect for (0.66 < Choen’s d < 1.00), or very large effect (d > 1.00). # Welch’s *t*-test.

## Data Availability

The data presented in this study are available on request from the corresponding author. The data are not publicly available due to privacy and ethical reasons.
